# Type IV Dual Left Anterior Descending Coronary Artery:A Rare Anomaly

**DOI:** 10.4103/1995-705X.73214

**Published:** 2010

**Authors:** V. Bhatia, P. Arora, A. K. Pandey, U. Kaul

**Affiliations:** Fortis Hospital, Noida, Uttar Pradesh, India; Escorts Heart Institute and Research Centre and Fortis Hospital, Vasant Kunj, New Delhi, India

**Keywords:** Congenital, coronary anomaly, dual LAD

## Abstract

Type IV dual left anterior descending (LAD) coronary artery is a rare anomaly and was detected incidentally during a routine coronary angiogram. The article discusses the types of dual LAD and their clinical implications.

## CASE REPORT

**Figure 1:**
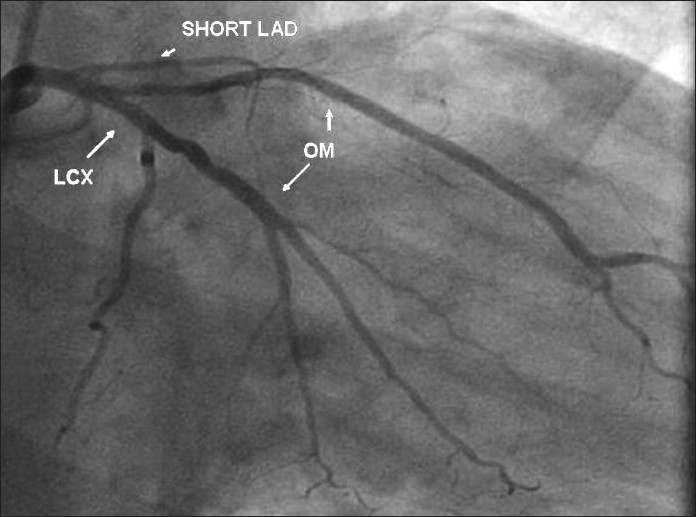
RAO view shows a short LAD; the left circumflex is normal

**Figure 2:**
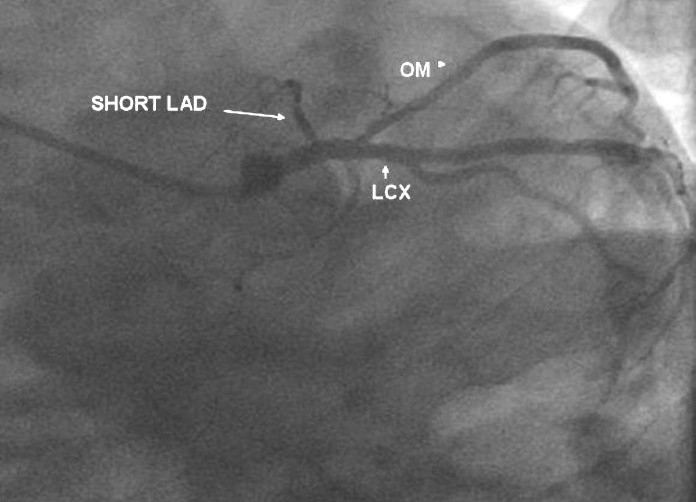
LAO cranial view shows a short LAD with small septal branches arising from the same. The left circumflex is normal

**Figure 3:**
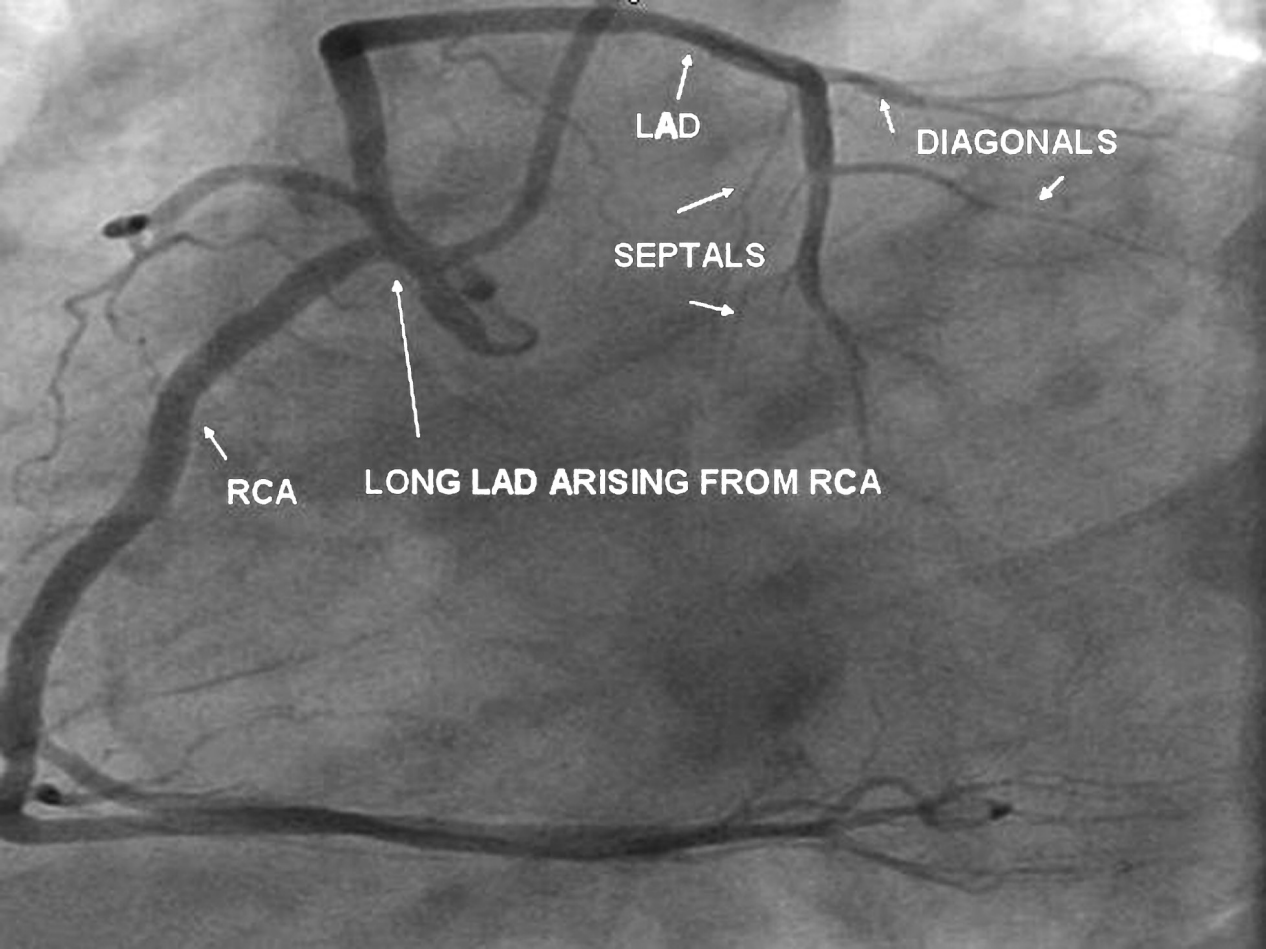
LAO view shows normal RCA. A long LAD is seen arising from the proximal RCA. Septals and diagonals are arising from the long LAD

A 56-year-old male, chronic smoker, diabetic, and hypertensive of 15-year duration was admitted with biventricular failure. His ECG revealed poor R-wave progression in anterior chest leads and echocardiogram was suggestive of global left ventricular (LV) hypokinesia, depressed LV systolic function (LVEF = 30%), and moderate pulmonary hypertension. His biochemical parameters were within normal limits. The patient was given aggressive decongestive therapy and showed marked improvement. Before discharge, he was subjected to coronary angiography to rule out any ischemic etiology for LV dysfunction. At CAG, there was no obstructive coronary artery disease; however, the patient had incidental detection of a congenital coronary anomaly in the form of dual LAD origin. The left anterior descending (LAD) artery arising from the left sinus was short and gave rise to septal branches [Figures 1 and 2]. The circumflex (LCX) and right coronary artery (RCA) were normal. A second LAD was seen arising from the proximal RCA. This LAD was long and septal and diagonal branches were also seen arising from this long anomalously arising LAD [Figure 3]. This was consistent with the type IV variety of dual LAD as per the Spinaldo-Franco Classification (see discussion), a rare entity.

## DISCUSSION

Congenital coronary anomalies are rare and reported to occur in 0.64-1.3% of patients undergoing coronary angiography.[1] They may be associated with underlying congenital heart disease (CHD) or may be incidentally detected at coronary angiography. Anomalies involving the origin, course, and distribution of the LAD are rare, even though such variations are common with the RCA. Dual LAD (also known as dual anterior interventricular artery) had been reported to occur with an incidence of 1% by Morettin[2] as well as Spinaldo-Franco et al.[3] Dual LAD may be associated with CHD as the tetrology of fallot and transposition of great arteries where it has surgical importance at the time of corrective surgery.[4]

Spinaldo-Franco *et al*. have proposed an angiographic classification for dual LAD[5] as follows:

**Type I:** Running in the anterior interventricular sulcus (AIVS), the short LAD is generally the source of all the major proximal septal perforators. The long LAD also runs in the AIVS, descending on the left ventricular side of the AIVS, and then reentering the distal AIVS in order to reach the apex.

**Type II:** The short LAD is the same as in Type I. The long LAD descends over the right ventricular side before reentering the AIVS.

**TypeIII:** The short LAD is consistent with that described in Types I and II. The long LAD travels intramyocardially in the ventricular septum.

**Type IV:** High in the AIVS, a very short vessel is formed by the LAD proper and the short LAD. From this vessel, the major septal perforators, as well as the diagonal branches, originate. The long LAD is unusual in its origin, arising from the RCA.

The presence of dual LAD has very little clinical significance in the absence of stenosis. When involved by obstructive disease, patients may be subject to revascularization percutaneously by surgery. Sajja et al. has stressed upon the fact that familiarity with dual LAD variants can help the surgeon avoid an incorrectly placed arteriotomy.[4]
